# Virus-Induced Maternal Immune Activation as an Environmental Factor in the Etiology of Autism and Schizophrenia

**DOI:** 10.3389/fnins.2022.834058

**Published:** 2022-04-12

**Authors:** Aïcha Massrali, Dwaipayan Adhya, Deepak P. Srivastava, Simon Baron-Cohen, Mark R. Kotter

**Affiliations:** ^1^Department of Clinical Neurosciences, University of Cambridge, Cambridge, United Kingdom; ^2^Autism Research Centre, Department of Psychiatry, University of Cambridge, Cambridge, United Kingdom; ^3^Department of Basic and Clinical Neuroscience, King’s College London, London, United Kingdom

**Keywords:** autism spectrum conditions, autism, maternal immune activation (MIA), SARS-CoV-2, schizophrenia, LPS, Poly(I:C)

## Abstract

Maternal immune activation (MIA) is mediated by activation of inflammatory pathways resulting in increased levels of cytokines and chemokines that cross the placental and blood-brain barriers altering fetal neural development. Maternal viral infection is one of the most well-known causes for immune activation in pregnant women. MIA and immune abnormalities are key players in the etiology of developmental conditions such as autism, schizophrenia, ADHD, and depression. Experimental evidence implicating MIA in with different effects in the offspring is complex. For decades, scientists have relied on either MIA models or human epidemiological data or a combination of both. MIA models are generated using infection/pathogenic agents to induce an immunological reaction in rodents and monitor the effects. Human epidemiological studies investigate a link between maternal infection and/or high levels of cytokines in pregnant mothers and the likelihood of developing conditions. In this review, we discuss the importance of understanding the relationship between virus-mediated MIA and neurodevelopmental conditions, focusing on autism and schizophrenia. We further discuss the different methods of studying MIA and their limitations and focus on the different factors contributing to MIA heterogeneity.

## Introduction

Maternal immune activation (MIA) is a major environmental factor known to increase likelihood of neurodevelopmental conditions such as autism (Paraschivescu et al., 2020) and schizophrenia (Kepinska et al., 2020; Purves-Tyson et al., 2021). MIA is mediated by activation of inflammatory pathways resulting in increased levels of cytokines and chemokines that cross the placental and blood-brain barriers (Patel et al., 2020; Mueller et al., 2021). This surge of inflammatory molecules alters neurodevelopment in the fetus (Patel et al., 2020; Purves-Tyson et al., 2021).

A number of maternal conditions may be responsible for triggering this inflammatory response, including autoimmune conditions (Chen et al., 2016), and asthma and allergic conditions (Gong et al., 2019). However, inflammatory responses caused by maternal viral infections remains one of the major environmental causes of MIA (Careaga et al., 2017; Baines et al., 2020; Cheslack-Postava and Brown, 2021). Case studies have associated viral infection from varicella, cytomegalovirus, mumps and herpes simplex virus with increased likelihood of autism (Patterson, 2011; Estes and McAllister, 2015). Understanding of the effects of virus-mediated MIA have also led researchers to consider the potential effects of maternal SARS-CoV-2 infection on fetal development (Reyes-Lagos et al., 2021).

## Investigating the Effects of Maternal Immune Activation on Fetal Brain Development

Studying the effects of viral-induced MIA on the offspring can be very challenging. For decades, scientists relied on either MIA models or human epidemiological data or a combination of both.

### Maternal Immune Activation Presentation From Human Epidemiological Studies

Epidemiological data has long implicated MIA as a likelihood factor for neurodevelopmental and neuropsychiatric conditions (Brown et al., 2004, 2014; Brown and Derkits, 2010; Estes and McAllister, 2016). More than 30 years ago an observation of higher incidence of schizophrenia and autism likelihood in winter/spring birth encouraged further investigations of specific infections such as influenza (Mednick et al., 1988; Kendell and Kemp, 1989). Earlier, human studies suggested associations between influenza and schizophrenia relying on ecological data, which focused on exposure to influenza epidemics in Europe, Australia and Japan (McGrath and Castle, 1995; Morgan et al., 1997; Izumoto et al., 1999; Mino et al., 2000). While some investigations reported an association between schizophrenia and infection during the second trimester of pregnancy (Mednick et al., 1988, 1994; Barr et al., 1990; Takei et al., 1996; Limosin et al., 2003), other studies reported conflicting results (Erlenmeyer-Kimling et al., 1994; Takei et al., 1995; Morgan et al., 1997; Westergaard et al., 1999). The initial study (Murray and Lewis, 1987) was met with a lot of criticism, as it failed to account for later onset schizophrenia and post adolescence changes (Folsom et al., 2006; Kochunov and Hong, 2014). Upon further investigations, studies suggest the “three-hit” model or “multiple hit” theory, making environmental, genetic, and maternal infections (e.g., influenza) potential hits (Keshavan, 1999; Davis et al., 2016). This theory also proposed that maternal infection increased the risk for childhood infections and later in life schizophrenia development (Blomström et al., 2016). Other studies looked at increased likelihood of schizophrenia with other types of infections such as measles (Fuller Torrey et al., 1988), mumps (O’Callaghan et al., 1994), and varicella zoster infections (Fuller Torrey et al., 1988; O’Callaghan et al., 1994), however, the results were conflicting and inconclusive. The inconsistency of the findings reported may in part be due to methodological limitation (Brown and Meyer, 2018). Ecological studies define exposure according to population-level prevalence at a specific location and a defined time and do not assess individuals in terms of actual infection evidence. More refined methods have emerged since then such as birth cohorts or case-control designs. Birth cohorts prospectively acquire serologic biomarkers of infection during individual pregnancies and investigate their association with higher likelihood of schizophrenia, autism and bipolar disorder (Meyer et al., 2009a; Brown and Derkits, 2010; Harvey and Boksa, 2012; Meyer, 2014; Estes and McAllister, 2016; Oliveira et al., 2017).

Studying MIA in humans relies on epidemiological data and does not investigate the potential biological pathways. The use of animal models filled this gap and provided the tool to examine the effects of MIA on fetal brain development.

### Maternal Immune Activation Animal Models Reveal Underlying Neurodevelopmental Mechanisms

The first animal model to study the effects of viral infection-induced MIA on the offspring was developed by exposing a pregnant mouse to the human influenza virus (Fatemi et al., 2004). Studies have also used prenatal administration of immunogenic liposaccharides (LPS), a cell wall component of a gram-negative bacteria that induces an immunogenic reaction *via* the transmembrane protein toll-like receptor (TLR) 4, or polyriboinosinic–polyribocytidilic acid [Poly(I:C)] which is a synthetic analog of dsRNA and induces an immunogenic reaction in rodents *via* the TLR3 (Shi et al., 2003; Fatemi et al., 2004; Jurgens et al., 2012; Honda-Okubo et al., 2014; Xia et al., 2014). Administering these elements to pregnant rodents (Meyer et al., 2005; Saadani-Makki et al., 2008; Meyer, 2014) and to non-human primates (Bauman et al., 2013; Machado et al., 2015; Weir et al., 2015; Rose et al., 2017) triggers the maternal immune system, resulting in the secretion of pro-inflammatory cytokines, microglial activation, and the induction of pro-inflammatory transcription factors in the neonatal brain.

The effects of this activation on fetal brain development may vary according to multiple factors including the timing of infection and the resilience of the developing brain, increasing the likelihood of developing neurodevelopmental conditions later in life (Meyer, 2014). For instance, earlier studies reported that prenatal exposure to Poly(I:C) leads to brain histopathological features in the offspring similar to those reported in schizophrenia, such as decreased hippocampal, prefrontal, cortical and striatal volume, and enlarged ventricles (Zuckerman et al., 2003; Piontkewitz et al., 2011). Other studies reported that prenatal infections is associated with reduced Purkinje neurons in the cerebellum, commonly reported in autism cases (Shi et al., 2009; Naviaux et al., 2013).

In rodents, exposure to Poly(I:C) for a specific time course (48 h) is sufficient to produce an acute inflammatory response with elevated individual cytokine levels (Cunningham et al., 2007; Fortier et al., 2007).

Some studies showed that irreversible neurodevelopmental defects in rodents induced by Poly(I:C) are dependent on IL-6 and IL-17A induced by MIA (Choi et al., 2016; Smith et al., 2016; Wu et al., 2017). For instance, a single maternal injection of IL-6 on day 12.5 of mouse pregnancy causes pre-pulse inhibition (PPI) and latent inhibition (LI) deficits in the adult offspring, mimicking central features of schizophrenia (Smith et al., 2007). Moreover, even when introduced externally, IL-6 is sufficient to alter brain development in the offspring (Ponzio et al., 2007; Smith et al., 2007). IL-6 is known for its regulatory role in self-renewal among neuronal precursor cells, neuronal migration, and neurite outgrowth (Goines and Ashwood, 2013). IL-6 is also a key factor for T helper cells (T_H_17) differentiation in both human and mice. IL-17A, the main T_H_17 cytokine has been found elevated in the serum of some children with autism (Suzuki et al., 2011; Al-Ayadhi and Mostafa, 2012). Choi et al. (2016), investigated the effect of the pathological activation of maternal IL-17A pathways on fetal development by pre-treating pregnant mothers with IL-17A blocking antibodies before injecting them with poly(I:C) and examining cortical development in the fetus. They reported disorganized cortical phenotypes in offspring following *in utero* MIA and autism-like behavioral abnormalities in offspring (Choi et al., 2016).

Another cytokine that has been associated with autism-like behavioral changes in the mice offspring is IL-2 (Ponzio et al., 2007). Interestingly, the behavioral changes reported after the dual administration of LPS and Poly(I:C) were not seen when co-administering antibodies for IL-6 and IL-2, which demonstrates that the biological, structural, and behavioral changes are mediated by cytokines (Smith et al., 2007; Girard et al., 2010).

Collectively, these studies show that these pro-inflammatory cytokines (resulting from maternal infection) alter fetal brain development. The exact mechanism through which these cytokines affect the brain and increase the likelihood of neurodevelopmental conditions is not clear, however, one theory is that the maternal induced activation resulting from prenatal infections lead to alterations in immunogenic molecules known to regulate neuronal function in the offspring (Coiro et al., 2015; Estes and McAllister, 2015).

#### Limitations of MIA Models

Maternal immune activation models provide an invaluable experimental tool in investigating the link between maternal infection and inflammatory molecules and altered fetal brain development outcomes and likelihood of neuropsychiatric conditions (Meyer et al., 2009a,b; Harvey and Boksa, 2012; Meyer, 2014). Nevertheless, these models have certain limitations. First, the induction of a maternal immune response using non-virulent agents [such as LPS and Poly(I:C)] cannot reproduce the full spectrum of immune response that would normally result following an infectious pathogen. This method does not recapitulate pathogen-specific humoral and cellular immune reaction, which is crucial in understanding the specific mechanism contributing to the potential association (Shi et al., 2003; Meyer et al., 2005). Although it has been shown that the outcomes associated with MIA are not pathogen-specific but rather are mediated by immune molecules (e.g., cytokines) triggered by various infections, it is nonetheless important to note that the mechanisms mediating these responses are specific to the pathogen (Gilmore and Jarskog, 1997; Shi et al., 2003; Meyer et al., 2009b; Labouesse et al., 2015).

Additionally, the controlled nature of the environment in which the experiments are usually set up excludes the real-life influences in humans. These influences are important contributors to the susceptibility and resilience to maternal infection, influencing the outcomes (Meyers, 2019).

Lastly, the use of rodents as MIA animal models might sometimes be misleading and the field might benefit by expanding studies to include more species that are evolutionarily and ethologically closer to humans (Phillips et al., 2014; Bauman and Schumann, 2018).

Rhesus macaques can provide a model to replicate the findings found in rodents MIA models as they exhibit greater similarity to humans regarding gestational timelines and fetal brain development (Short et al., 2010; Phillips et al., 2014; Bauman and Schumann, 2018). For instance, Short et al. (2010) investigated the effects of immune activation during the third human trimester immune, which cannot be possible in rodents whose equivalent developmental stages occur postnatally. Moreover, Rhesus macaque MIA studies offer another benefit over rodents which is the ability to measure social behavioral phenotypes, such as social attention and detection of facial expressions (Machado et al., 2015). Other studies rely on induced pluripotent stem cells to advance our understanding of how specific cytokines induced by MIA can increase the likelihood of developing neurodevelopmental conditions (Warre-Cornish et al., 2020).

## Heterogeneity in Maternal Immune Activation

Exposure to infections during pregnancy is quite common; almost 50% of pregnant women get respiratory tract infections while close to 20% getting urinary tract infections; nonetheless this high prevalence of maternal infection results in neurodevelopmental abnormalities in only a small portion of the exposed offspring (Milada et al., 2017; Weber-Stadlbauer, 2017; Brown and Meyer, 2018). This dichotomy might hold the key to certain protective that make certain pregnancies less susceptible to others, and mechanistically evidence has suggested impairment of cholinergic anti-inflammatory pathways in some pregnant mothers to be linked to a heightened inflammatory response (Reyes-Lagos et al., 2021).

Positive associations have been reported between maternal proinflammatory molecules [(IL)-1a, IL-6, IL-8, interferon-gamma (IFN-g), tumor necrosis factor alpha (TNF-a), granulocyte macrophage colony-stimulating factor (GMCF), and C-reactive protein (CRP)] and increased likelihood of autism and schizophrenia. Other studies looked at the effects of increased levels of maternal cytokines on fetal brain development, and reported alterations in the connectivity of the amygdala (Graham et al., 2018; Rudolph et al., 2018), and frontolimbic white matter (Rasmussen et al., 2019) and observed cognitive abnormalities in toddlers (Graham et al., 2018; Rudolph et al., 2018; Rasmussen et al., 2019). These studies have confirmed the link between maternal infections, the role of abnormal levels of cytokines and the possibility of developmental anomalies in the offspring (Brown and Meyer, 2018; Gumusoglu and Stevens, 2019; Weber-Stadlbauer and Meyer, 2019). Notwithstanding the abundance of evidence implicating MIA in the etiology of neuropsychiatric conditions, this pathogenic model appears to be naïve. The type of infection, gestational time and the resilience of the maternal immune system are important factors to consider when trying to dissect the heterogeneity of the MIA-induced effects (Meyers, 2019).

Identifying the factors that promote resilience to the MIA or vulnerable factors that might make some pregnancies more susceptible is of crucial importance. Different maternal infections have distinct mode of actions even if the effect associated is dependent on the immune reaction and not the type of infection. Some pathogens can directly affect the offspring through vertical transmission from the mother to the fetus through the placenta or through breast milk. Other pathogens are non-transmissible from the mother to the fetus and they still impose serious influences on the offspring for neurodevelopmental and neuropsychiatric conditions (Mednick et al., 1988; Milada et al., 2017; Brown and Meyer, 2018). The specificity of the proinflammatory modulators produced post infection can influence the severity of MIA effects on fetal brain developments, is the specificity of the proinflammatory modulators produced post infection. For instance, recent studies using animal MIA models report that different subtypes of toll-like receptors produce different outcomes in the offspring (Meyer, 2014; Weber-Stadlbauer and Meyer, 2019). Prenatal activation of TLR3 resulted in cellular, neurochemical, and behavioral phenotypes of a hyperdopaminergic state (Luan et al., 2018) while prenatal activation of TLR4 induced a hypodopaminergic state (Kirsten et al., 2012). This supports the notion that different immunological molecules might have different pathological outcomes. The intensity and timing of infections are potential contributors to the susceptibility of certain pregnancies to MIA. Studies have shown a positive correlation between the severity of maternal inflammation and anomalies in the developing brain in the general population and in a schizophrenia cohort (Graham et al., 2018; Rudolph et al., 2018; Rasmussen et al., 2019). Similarly, dose-dependent effects have been confirmed in animal models of MIA (Meyer et al., 2005; Hornig et al., 2018).

Maternal exposure to TORCH pathogens imposes a higher probability of developing neurodevelopmental anomalies in the first half of the pregnancy while the effects of the infection are subtle and less severe in later gestational periods. Similarly, birth cohort studies suggested a trimester -dependent effects of MIA in increasing autism incidence (Hornig et al., 2018). Maternal micronutrients such as vitamin D, iron, zinc omega-3 fatty acids and choline and others have also been identified as important factors to promote resilience to infections and optimal immune functioning (Brown, 2011; Luan et al., 2018; Maggini et al., 2018; Mattei and Pietrobelli, 2019).

The microbiome has been recently added to the equation as a potential etiological factor not only in the neurodevelopmental condition but also in the dysregulation of immune functions (Conway and Brown, 2019). Therefore, it is one of the most crucial factors in MIA heterogeneity. Immune function abnormalities have been reported in autism and other neuropsychiatric conditions, and recent studies propose the involvement of the microbiota in this dysregulation (Hsiao et al., 2013; Cryan and Dinan, 2015; Erny et al., 2015). Experimental studies looking at the immunological effects of MIA reported dysregulation of offspring microbiota in adulthood (Hsiao et al., 2013; Mandal and Ghosh, 2013; Kim et al., 2017). A 2017 study found that mice that have more T_H_17 cells with segmented filamentous bacteria (SFB) were more susceptible to produce behavioral changes caused by MIA (Kim et al., 2017). Both MIA and microbiome dysregulation have been associated with alterations in fetal brain development, autism, and other neurodevelopmental conditions. Moreover, MIA activation alters maternal gut bacteria which can affect the microbiome of the offspring (Conway and Brown, 2019). This makes it extremely challenging to dissect the cause-effect relationship between MIA and the maternal microbiota, on offspring neurodevelopment.

Finally, the genetic background is a crucial contributor to the susceptibility or resilience to neurodevelopmental effect of MIA. Recent epidemiological studies have reported that familial history of psychiatric conditions posits synergistic interaction with MIA in increasing the probability of later developing schizophrenia. However, studies that examining common variants associated with schizophrenia showed no correlation interaction with MIA in increasing the likelihood of schizophrenia in the offspring. The latter does not remove the genetic background as a potential contributor to MIA heterogeneity but rather points out that polygenic scores are not proxy for genetic contribution (Clarke et al., 2009; Nielsen et al., 2013; Benros et al., 2016; Blomström et al., 2016). The genetic factors can be divided into factors that promote MIA susceptibility (Ayhan et al., 2016) or protection (Meyer et al., 2008). For instance, IL-10 polymorphisms have been associated with increased resilience against MIA effects as the increased production of IL-10 offer neurodevelopmental protection in the offspring post maternal infection (Meyer et al., 2008).

## Potential Effects of SARS-COV-2 on Fetal Brain Development

SARS-CoV-2 infection is known to stimulate production of MIA-causing pro-inflammatory cytokines such as IL-6, IL10 and TNFα (Pedersen and Ho, 2020). Since the virus has only been identified recently (end of 2019) there are yet no studies that have been able to investigate neurodevelopmental consequences in the offspring of affected mothers. Based on its clinical manifestation and past “similar” infections, theories have been proposed that suggest potential effects on the offspring (Granja et al., 2021). Following infection with SARS-CoV-2, there is an uncontrolled release of inflammatory cytokines and chemokines that have been involved in brain development [e.g., interleukins (IL-1β,−2,−4,−6,−8,−10), tumor necrosis factor alpha, interferons (IFN-α, IFN-γ)] (Reyes-Lagos et al., 2021). Amid the exacerbated long-term inflammatory effects observed in COVID-19 patients and the lessons learned from of viral-mediated MIA effects on the progeny’s brain, it is crucial to consider the neurological consequences of maternal SARS-CoV-2 infection on the offspring.

## Conclusion

Human epidemiological data and MIA animal models have consistently shown MIA is associated to increased likelihood of developmental neuropsychiatric conditions; however, the effects are very heterogenous. The high percentage of infected women that give birth to neurotypical offspring highlight that one pregnancy may be more susceptible to MIA adverse outcomes than the other. Studies to identify these factors aim to protect fragile pregnancies and inform us on potential contributors to the development of neurodevelopmental conditions.

## Author Contributions

AM wrote the manuscript with support from DA and in consultation from DS, SB-C, and MK who supervised and provided critical feedback that shaped the final version of the manuscript.

## Author Disclaimer

Any views expressed are those of the author(s) and not necessarily those of the funder.

## Conflict of Interest

The authors declare that the research was conducted in the absence of any commercial or financial relationships that could be construed as a potential conflict of interest.

## Publisher’s Note

All claims expressed in this article are solely those of the authors and do not necessarily represent those of their affiliated organizations, or those of the publisher, the editors and the reviewers. Any product that may be evaluated in this article, or claim that may be made by its manufacturer, is not guaranteed or endorsed by the publisher.
