# Corrigendum: Virus-Induced Maternal Immune Activation as an Environmental Factor in the Etiology of Autism and Schizophrenia

**DOI:** 10.3389/fnins.2022.943903

**Published:** 2022-06-08

**Authors:** Aïcha Massrali, Dwaipayan Adhya, Deepak P. Srivastava, Simon Baron-Cohen, Mark R. Kotter

**Affiliations:** ^1^Department of Clinical Neurosciences, University of Cambridge, Cambridge, United Kingdom; ^2^Autism Research Centre, Department of Psychiatry, University of Cambridge, Cambridge, United Kingdom; ^3^Department of Basic and Clinical Neuroscience, King's College London, London, United Kingdom

**Keywords:** autism spectrum conditions, autism, maternal immune activation (MIA), SARS-CoV-2, schizophrenia, LPS, Poly(I:C)

In the original article, an author's name was incorrectly spelled as Aïcha Massarali. The correct spelling is Aïcha Massrali.

The authors apologize for this error and state that this does not change the scientific conclusions of the article in any way. The original article has been updated.

## Publisher's Note

All claims expressed in this article are solely those of the authors and do not necessarily represent those of their affiliated organizations, or those of the publisher, the editors and the reviewers. Any product that may be evaluated in this article, or claim that may be made by its manufacturer, is not guaranteed or endorsed by the publisher.

